# Retraction: Paraneoplastic IgA Vasculitis in an Older Adult With Metastatic Lung Adenocarcinoma

**DOI:** 10.7759/cureus.r37

**Published:** 2021-12-06

**Authors:** Khashayar Farzam, Harris Syed

**Affiliations:** 1 Family Medicine, University of Iowa Hospitals and Clinics, Iowa City, USA

This article has been retracted at the request of the submitting author, Khashayar Farzam, as the University of Iowa Hospitals & Clinics patient consent procedure was not properly followed by the authors when preparing this manuscript. Written consent was required from the patients, but only verbal consent was obtained. Due to the rarity of this condition and the inclusion of specific case details, the article content has been removed in order to avoid any potential privacy violations. The authors would like to apologize for their mistake as they believed that verbal consent was sufficient.

